# Childhood Acute B-Lineage Lymphoblastic Leukemia With CDKN2A/B Deletion Is a Distinct Entity With Adverse Genetic Features and Poor Clinical Outcomes

**DOI:** 10.3389/fonc.2022.878098

**Published:** 2022-05-24

**Authors:** Jing Feng, Ye Guo, Wenyu Yang, Yao Zou, Li Zhang, Yumei Chen, Yingchi Zhang, Xiaofan Zhu, Xiaojuan Chen

**Affiliations:** State Key Laboratory of Experimental Hematology, National Clinical Research Center for Blood Diseases, Haihe Laboratory of Cell Ecosystem, Institute of Hematology & Blood Diseases Hospital, Chinese Academy of Medical Sciences & Peking Union Medical College, Tianjin, China

**Keywords:** *CDKN2A/B*, pediatric acute lymphoblastic leukemia, fluorescence *in situ* hybridization, prognosis, *TP53*

## Abstract

To further emphasize the clinical–genetic features and prognosis of *CDKN2A/B* deletions in childhood acute lymphoblastic leukemia (ALL), we retrospectively analyzed 819 consecutive B-ALL patients treated with the Chinese Children’s Cancer Group ALL-2015 (CCCG-ALL-2015) protocol, and fluorescence *in situ* hybridization (FISH) analysis on *CDKN2A/B* deletion was available for 599 patients. The prevalence of *CDKN2A/B* gene deletions was 20.2% (121/599) of B-ALL. *CDKN2A/B* deletions were significantly associated with older age, higher leukocyte counts, a higher percentage of hepatosplenomegaly, and a higher frequency of *BCR-ABL* (*p* < 0.05). Those patients achieved similar minimal residual disease (MRD) clearance and complete remission compared to patients without *CDKN2A/B* deletion. The *CDKN2A/B* deletions were correlated with inferior outcomes, including a 3-year event-free survival (EFS) rate (69.8 ± 4.6 vs. 89.2 ± 1.6%, *p* = 0.000) and a 3-year overall survival (OS) rate (89.4% ± 2.9% vs. 94.7% ± 1.1%, *p* = 0.037). In multivariable analysis, *CDKN2A/B* deletion was still an independent prognostic factor for EFS in total cohorts (*p* < 0.05). We also detected a multiplicative interaction between *CDKN2A/B* deletions and TP53 deletion on dismal prognosis (*p*-interaction < 0.05). In conclusion, *CDKN2A/B* deletion is associated with distinct characteristics and serves as a poor prognostic factor in pediatric ALL, especially in TP53 deletion carriers.

## Introduction

Pediatric acute lymphoblastic leukemia (ALL) is one of the most curable malignancies, with 5-year event-free survival rates exceeding 80% in many developed countries and even exceeding 90% in high-income countries (1). With the increased understanding of genetic alterations in ALL, many molecular markers have been identified and applied to risk stratification and treatment protocols in leukemia. *CDKN2A/B* is one of the most frequent abnormal genes in ALL and can be detected by fluorescence *in situ* hybridization (FISH), multiplex ligation-dependent probe amplification (MLPA), array-based comparative genomic hybridization (aCGH) analysis, and single-nucleotide polymorphism array (SNPA) (2–6). As a secondary genetic event in the development of leukemia, the *CDKN2A/B* deletions were found in approximately 20%–25% of B-cell precursor acute lymphoblastic leukemia (BCP-ALL) and 38.5%–50% of T-ALL patients (2, 5, 7, 8). Despite the high frequency of *CDKN2A/B* deletions in pediatric ALL, the prognostic importance of the deletions is still inconclusive. Most investigators have concluded that the deletions were associated with the recurrence of pediatric ALL (7, 9, 10), and some researchers found that the inactivation of *CDKN2A/B* did not influence the outcome of childhood B-lineage ALL (4, 11). As previous inconsistent conclusions were drawn from small cohorts, large sample-sized studies are needed to clarify the prognostic impact of *CDKN2A/B* deletion in pediatric ALL. In this study, we assessed the clinical and biological characteristics and prognostic factors of *CDKN2A/B* deletions in 662 pediatric ALL patients.

## Materials and Methods

### Patients and Treatment Protocols

**Figure 1:**
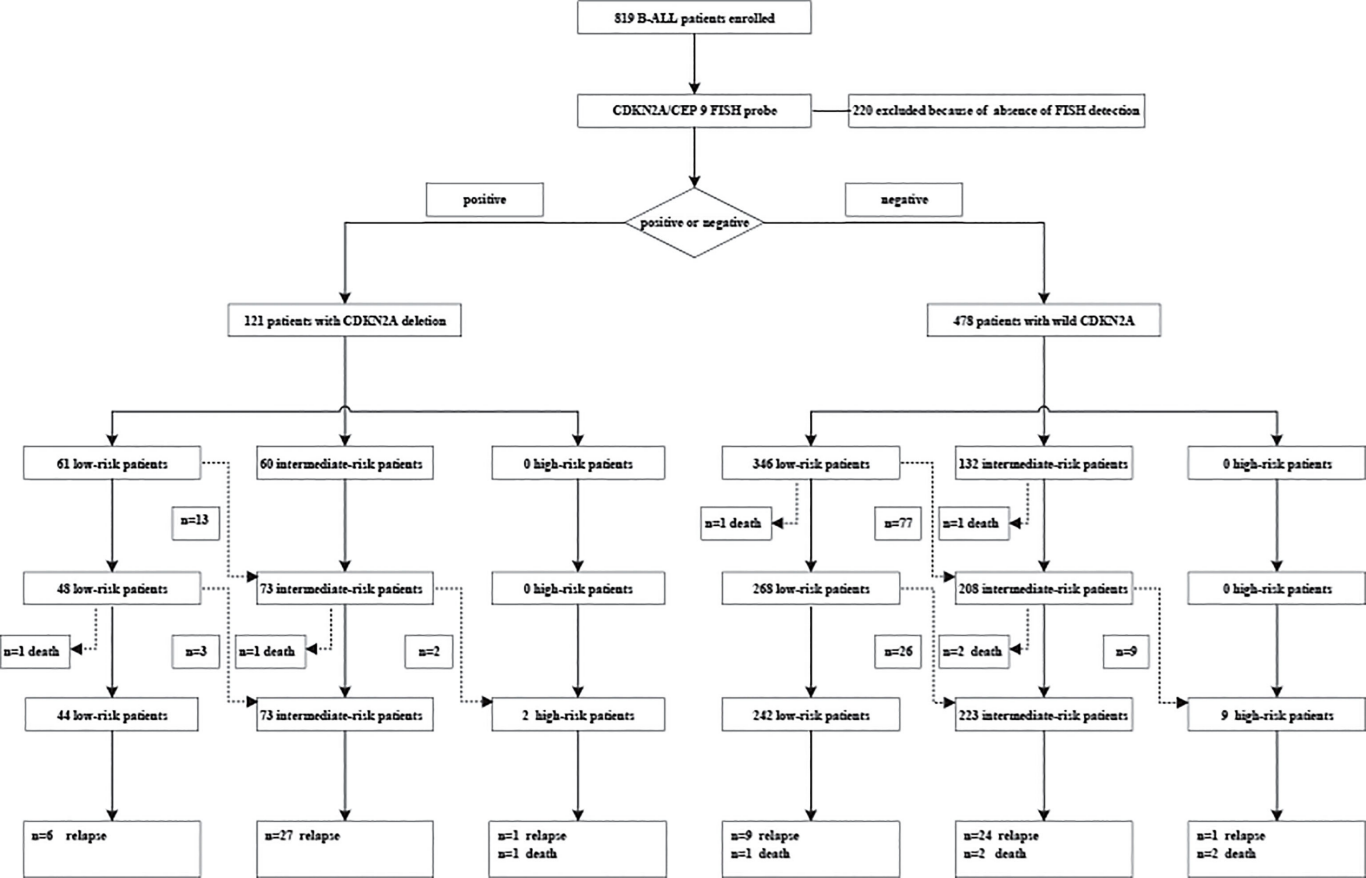
Clinical profile.

The cohort included 902 patients with newly diagnosed pediatric ALL who were treated according to the CCCG-ALL 2015 protocol (ClinicalTrials.gov identifier: ChiCTRIPR-14005706) (12) registered at the Blood Disease Hospital of CAMS & PUMC between May 2015 and December 2019. As the FISH test has been performed since September 2016, FISH data from 662 patients were collected in this cohort finally (**Figure 1**). The protocol described in this study was approved by the Ethics Committee, Institute of Hematology and Blood Disease Hospital, Diseases Hospital, Chinese Academy of Medical Sciences (CAMS) and Peking Union Medical College (PUMC) (No. IIT2015010-EC-1). All patients or their legal guardians signed written informed consent before treatment.

### Risk Group Assignment

Patients enrolled in the CCCG-ALL-2015 study were assigned to different risk groups based on morphology and immunophenotypic and genetic features of leukemia cells. Patients were assigned to the low-risk group if they had B-cell ALL and were aged between 1 year and 10 years; had a leukocyte count of less than 50 × 10^9^/L, a chromosome number of more than 50, or the ETV6–RUNX1 fusion gene; and did not have CNS 3 status or testicular leukemia, and had a minimal residual disease of less than 1% on day 19 of induction and less than 0.01% on day 46 of induction. Patients with a minimal residual disease of 1% or more (or ≥5% blasts morphologically without suitable markers for minimal residual disease) in bone marrow on day 46 of induction and infants younger than 6 months with KMT2A rearrangement and a leukocyte count of 300 × 10^9^/L or more were classified as high-risk ALL. The remaining participants were assigned to the intermediate-risk group (12). All eligible patients received minimal residual disease-directed, risk-stratified treatment modified from the St Jude Children’s Research Hospital Total Therapy 15 and 16 studies (13, 14) and the Shanghai Children’s Medical Center ALL-2005 trial (15), with IR/HR patients receiving more intensive treatment.

### FISH and Probe

Pretreatment bone marrow aspirates were taken at diagnosis and at least 1–2 ml of bone marrow aspirates was analyzed by FISH for cytogenetic abnormalities (including CDKN2A/B, KMT2A rearrangement, TP53 deletion, and BCR-ABL1), polymerase chain reaction (PCR) for fusion gene (including ETV6-RUNX1, BCR-ABL, and TCF3-PBX1) (16), and karyotyping. We analyzed interphase cells according to the instructions of the probe manufacturer (America Abbott). The *CDKN2A/B* probe spanned approximately 222 kilobases (kb) and contained many genes, including methylthioadenosine phosphorylase, *CDKN2A* (*INK4A* and *ARF*), and *CDKN2B* (*INK4B*) in the 9p21 chromosome region. The cutoff level for positive results was calculated to be 5%, and at least five hundred cells were analyzed (5, 17). Some cases with two different deleted populations (one biallelic and one monoallelic) were classified as having a biallelic deletion.

### Early Treatment Response Definitions and Endpoints

To identify early prognostic factors, we evaluated leukemic blast counts in bone marrow by morphology on day 46 and minimal residual disease (MRD) on days 19 and 46 during the first induction. A total of 1–2×10^6^ leukocytes from bone marrow were incubated with marker panel [B-lineage cells (CD10, CD19, TdT, cyμ, sIgM, CD20, cyCD22, CD22, and cyCD79a), T-lineage cells (CD1a, CD2, CD3, CD4, CD5, CD7, CD8, TCRαβ, TCRγδ, and cyCD3), or myeloid cells (CD11b, CD13, CD14, CD15, CD33, CD41, CD61, CD64, CD65, CD71, GPA, and cyMPO)] for 30 min, washed twice, and then resuspended in 100 μl of PBS. The cells were acquired on a Navios (Beckman Coulter, UK) and analyzed using Kaluza software. Samples were defined as MRD-negative if no baseline and/or different-from-normal leukemia-associated immunophenotypes (LAIP) cells could be quantitated above the limit of detection (approximately 0.01%) (18).

Diagnosis of complete remission (CR) was based solely on bone marrow morphology with a cutoff value of 5% of leukemic blasts on treatment day 46. Bone marrow MRD that is not lower than 10^-5^ was defined as positive. A poor response to prednisone was considered if the peripheral blast count on treatment day 5 was not lower than 1×10^9^/L.

The primary endpoint event-free survival was calculated from the date of diagnosis until the following events: induction treatment failure, any relapse after CR, second malignancy, and death due to any cause. Overall survival was considered as the time from diagnosis to death due to any cause.

### Statistical Analysis

Chi-squared test and Mann–Whitney *U* tests were used to compare categorical and continuous variables, respectively. Event-free and overall survival were calculated and compared using Kaplan–Meier analysis and log-rank tests. Cox proportional hazard regression analyses were performed using univariable and multivariable regression approaches. Variables that were found statistically significant and a *p*-value of approximately 0.1 were included in multivariable Cox regression analysis. We performed subgroup analyses for prespecified baseline factors with rates of inferior events by factor interaction with the use of Cox models. Statistical significance was defined as a *p*-value of less than 0.05. All analyses were performed using SPSS v. 24. and SAS v. 9.4 software.

## Results

### Comparison of Demographic and Clinical Characteristics of Childhood ALL With and Without *CDKN2A/B* Deletions

In total, 599 patients were included in the analysis. The final follow-up was in December 2020 and the median follow-up time was 34 months (range: 0 to 58 months). The median age was 5 years (range, 0 to 14), and the male/female ratio was 1.4 (350/249). The prevalence of *CDKN2A/B* gene deletions in B-ALL was 20.2% (121/599). Among patients with *CDKN2A/B* deletions, 57 (45.3%) harbored *CDKN2A/B* biallelic deletions, whereas 82 (54.7%) harbored monoallelic deletions, and 11 (7.3%) patients harbored both biallelic and monoallelic deletions.

Compared to patients with wild-type *CDKN2A/B* (**Table 1**), patients with *CDKN2A/B* deletions were significantly associated with older age (age >10 years; 30.6% vs. 15.2%, *p* < 0.001), a higher leukocyte count (median: 24.7 vs. 8.9×10^9^/L, *p* < 0.001), a lower platelet count (median: 51 vs. 64 g/L, *p* = 0.049), and a higher percentage of hepatosplenomegaly (64.5% vs. 43.9%, *p* < 0.001). A higher rate of central nervous system status 2(CNS 2)/traumatic lumbar puncture was found among patients with *CDKN2A/B* deletion, but no statistical significance was found in the two groups. Patients with *CDKN2A/B* deletions belonged more to the intermediate-risk groups (58.7% vs. 32.6%, *p* < 0.01) than patients without *CDKN2A/B* deletions based on CCCG-ALL 2015 risk stratification. There was no significant difference between patients with biallelic deletions and monoallelic deletions. The comparison of characteristics for the two groups is shown in **Table S1**.

**Table 1:** Characteristics of B-ALL patients with CDKN2A/B deletion and CDKN2A/B wild type.

	Total	CDKN2A/B deletion (%)	CDKN2A/B wild type (%)	*p*-value
Number	599	121 (20.2)	478 (79.8)	
Gender, male/female	350/249	63/58	287/191	0.148
Age, ≥10 years	109 (18.2)	37 (30.6)	72 (15.2)	<0.001
Leukocyte counts, ×10^9^/L(range)	10.8 (0.7–846.5)	24.7 (0.7–846.5)	8.9 (0.74–551.2)	<0.001
Platelets, ×10^9^/L(range)	61 (1–919)	51 (3–503)	64 (1–919)	0.049
Hepatosplenomegaly	288 (48.1)	78 (64.5)	210 (43.9)	<0.001
CNS2/traumatic lumbar puncture	66 (11.0)	17 (14.1)	49 (10.3)	0.233
Risk stratification, IR	192 (32.1)	60 (49.6)	162 (33.9)	0.001
Cytogenetic abnormalities				
Chromosome number ≥50	47 (7.8)	6 (5.0)	41 (8.6)	0.186
Chromosome number <44	6 (1.0)	3 (2.5)	3 (0.6)	0.100
ETV6-RUNX1	152 (25.4)	24 (19.8)	128 (26.8)	0.117
t(9;22)(q34;q11.2)/BCR-ABL1	36 (6.0)	15 (12.4)	21 (4.4)	0.001
t(1;19)(q23;p13.3)/TCF3-PBX1	32 (5.3)	6 (5.0)	26 (5.4)	0.834
TP53 deletion	29 (4.8)	10 (8.3)	19 (4.0)	0.050
11q23/KMT2A	11 (1.8)	1 (0.8)	10 (2.1)	0.354
PPR	134 (22.4)	34 (28.1)	100 (20.9)	0.091
MRD positive on day 19	403 (67.7)	74 (61.2)	329 (69.4)	0.083
MRD positive on day 46	108 (18.3)	22 (18.5)	86 (18.2)	0.946
CR	593 (99.0)	119 (98.4)	474 (99.2)	0.350

### Correlation of *CDKN2A/B* Deletion With Other Cytogenetic Alterations

Karyotype analysis, 43 fusion genes, and FISH studies of pretreatment bone marrow were available for 599 patients. **Table 1** summarizes the correlations of *CDKN2A/B* deletions with other cytogenetic abnormalities. The *CDKN2A/B* deletion group had a higher prevalence of the *BCR/ABL* fusion gene (12.4% vs. 4.4%, *p* = 0.001). A higher co-occurrence of the *ETV6/RUNX1* fusion gene (19.8% vs. 26.8%) was found in patients with CDKN2A/B deletion; however, no statistical significance was identified. A higher incidence of chromosome < 44 (2.5% vs. 0.6%) and *TP53* deletions (8.3% vs. 4.0%) was detected by FISH, but this did not reach statistical significance (*p* ≥ 0.05). The genetic feature of patients with *CDKN2A/B* gene deletions was also related to the risk stratification distribution at the first diagnosis.

### The Effect of *CDKN2A/B* Gene Deletions on Early Treatment Responses

A chi-squared test showed a high rate of poor responses to prednisone (PPR) (28.1% vs. 20.9%, *p* = 0.091) in the CDKN2A/B deletion group. No significant difference was observed between the two groups for MRD on day 19 (61.2% vs. 69.4%, *p* = 0.946) and 46 (18.5% vs. 18.2%, *p* = 0.946). The CR rate (98.4% vs. 99.2%, *p* = 0.719) for each group was equivalent.

### Overall Outcomes

A high CR rate was achieved in 593 of 599 patients (99.0%) (**Figure 1**). Based on their MRD levels on days 19 and 46 during remission induction treatment, 286 (48.2%) of 593 patients were classified as having low-risk ALL, 296 (49.9%) as having intermediate-risk ALL, and 11 (1.9%) as having high-risk ALL. All six patients who did not achieve the first CR died of severe pneumonia. After remission induction, 74 patients had adverse events, including 68 relapses (*n* = 54 hematologic relapses, *n* = 4 combined hematologic and CNS relapses, *n* = 2 combined hematologic and testicular relapses, *n* = 1 combined hematologic, CNS, and testicular relapses, *n* = 4 isolated CNS relapse, *n* = 2 testicular relapse, and *n* = 1 ocular relapse). Six patients died [*n* = 2 died of severe pneumonia and emesis in remission and *n* = 4 died after chimeric antigen receptor T-cell immunotherapy (CAR-T) therapy or hematopoietic stem cell transplantation (HSCT) because of elevated MRD level]. The 3-year OS and EFS were 93.6 ± 1.1% and 85.2 ± 1.6%, respectively, for the whole series.

### The Effect of *CDKN2A/B* Deletions on the Prognosis of Pediatric ALL

**Figure 2:**
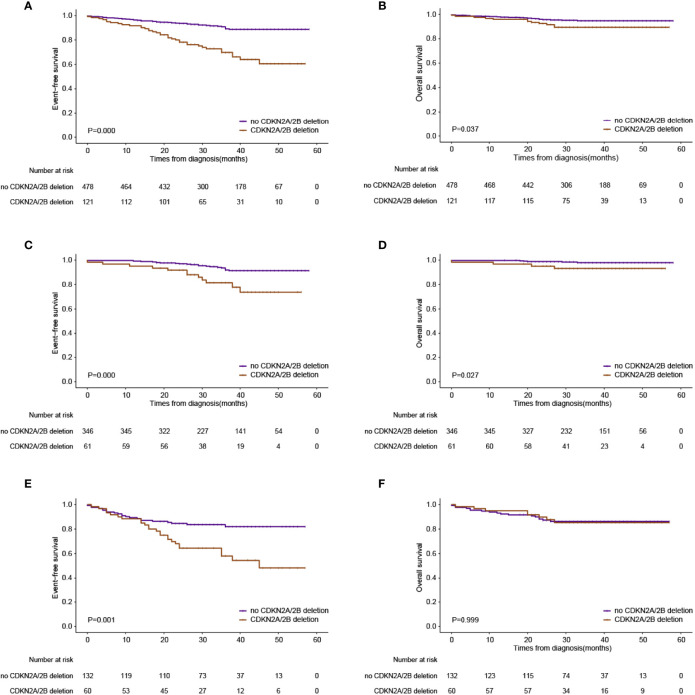
Outcomes based on the presence or absence of CDKN2A deletions. **(A)** Event-free survival in the whole series. **(B)** Overall survival in the whole series. **(C)** Event-free survival in the low-risk group. **(D)** Overall survival in the low-risk group. **(E)** Event-free survival in the intermediate-risk group. **(F)** Overall survival in the intermediate-risk group.

**Figure 3:**
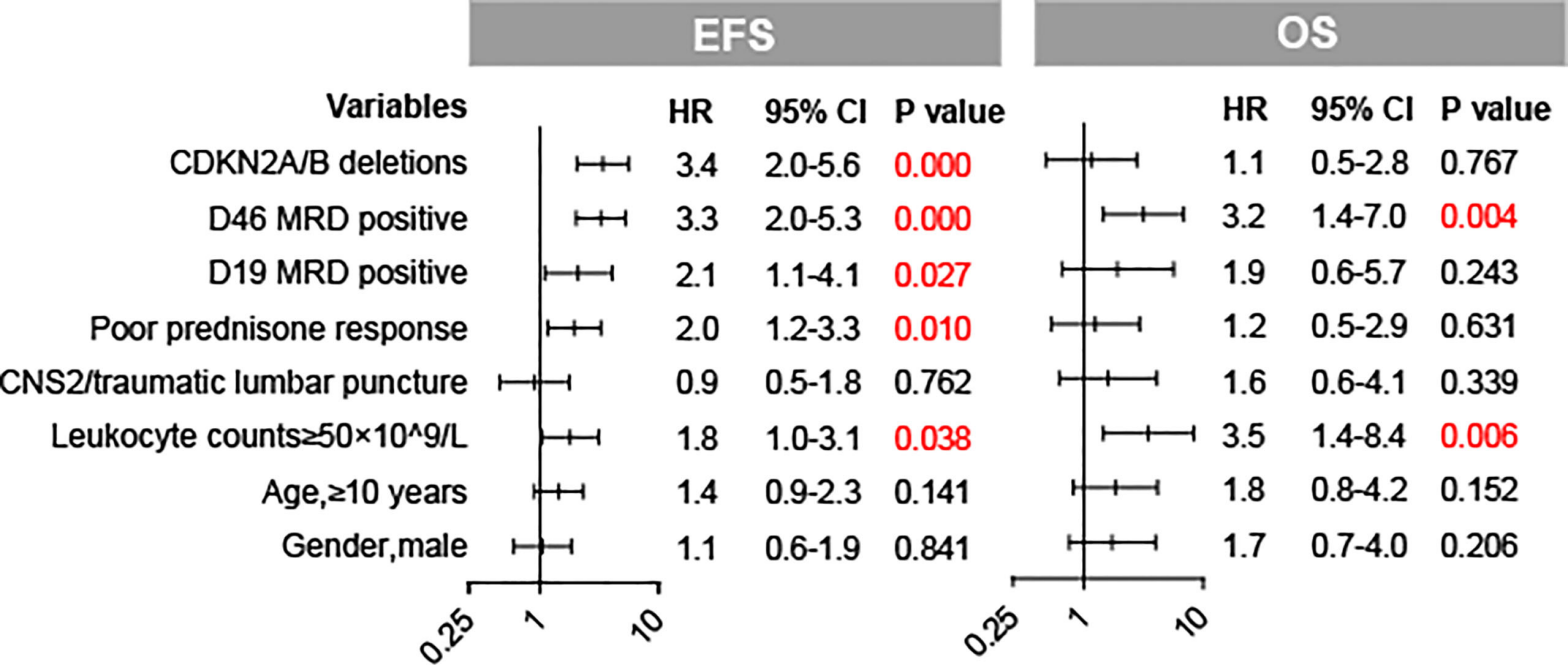
Forest plots of the multivariate analysis of CDKN2A/B deletions and baseline factors for EFS and OS in our cohort.

The *CDKN2A/B* deletion group is related to a higher relapse rate (28.1% for the deletions group vs. 7.1% for the wild-type group, *p* = 0.000) (**Table 2**). Patients with *CDKN2A/B* deletions seemed to have a higher CNS relapse rate than patients with *CDKN2A/B* deletions, although the *p*-value was not significant (3.3% vs. 1.1%, *p* = 0.087). Patients with *CDKN2A/B* deletions had lower 3-year EFS and OS rates than patients without *CDKN2A/B* deletions (3-year EFS: 69.8 ± 4.6 vs. 89.2 ± 1.6%, *p* = 0.000; and 3-year OS: 89.4% ± 2.9% vs. 94.7% ± 1.1%, *p* = 0.037) (**Figures 2A, B**). Furthermore, *CDKN2A/B* deletion serves as an independent prognosis factor (HR = 3.4 [95% CI 2.0–5.6]; *p* = 0.000) for EFS in whole cohorts (**Figure 3**). MRD on day 46 was a strong predictor (HR = 3.3 [95% CI 2.0–5.3]; *p* = 0.000) for all cases.

**Table 2:** Different types of relapse in all patients.

	Total(*n* = 599)	CDKN2A/B deletion (*n* = 121)	CDKN2A/B wild type (*n* = 478)	*p*-value	*χ* ^2^ value
Relapse	68 ()	34 (28.1)	34 (7.1)	0.000	42.258
Hematological relapse	61 ()	30 (24.8)	31 (6.5)	0.000	35.384
Any CNS relapse	9 ()	4 (3.3)	5 (1.1)	0.087	–
Isolated CNS relapse	4 ()	3 (2.5)	1 (0.2)	0.028	–
Any testicular relapse	5 ()	2 (1.7)	3 (0.6)	0.266	–

### Outcomes Stratified by Risk Groups and *CDKN2A/B* Deletions

On the basis of the CCCG-ALL 2015 risk stratification, more patients were classified as intermediate risk in the *CDKN2A/B* deletion group at diagnosis. For patients assigned into the low-risk group, patients with *CDKN2A/B* deletions had adverse clinical outcomes (3-year EFS: 81.4 ± 5.4% vs. 91.9 ± 1.7%, *p* = 0.000; 3-year OS: 93.2 ± 3.3% vs. 97.9 ± 0.9%, *p* = 0.027) compared to patients without *CDKN2A/B* deletions (**Figures 2C, D**). Intermediate-risk patients with *CDKN2A/B* deletions had inferior 3-year EFS (58.0 ± 7.1% vs. 82.0 ± 3.6%, *p* = 0.001), but no inferior 3-year OS (85.3 ± 4.9% vs. 86.3 ± 3.1%, *p* = 0.999) compared to intermediate-risk patients without *CDKN2A/B* deletions (**Figures 2E, F**).

### The Joint Effect of *CDKN2A/B* Deletions and Other Baseline Factors

We performed stratified analyses by subgroups defined by major co-variables that might have been related to EFS and further quantified the effect modification of major co-variables on the influence of *CDKN2A/B* deletions in EFS, accounting for important covariates (**Table 3**). The hazard rates of *CDKN2A/B* deletions in EFS were consistent in most subgroups. The hazard rate of *CDKN2A/B* deletions for the adverse events was quite different for patients with and without *TP53* deletions (HR = 24.080 [95% CI 2.978–194.738]; *p* = 0.0029 and HR = 2.993 [95% CI 1.868–4.796]; *p* < 0.0001, respectively), while there were no significant interactions between two groups (*p*-interaction = 0.0631).

**Table 3:** Hazard ratio for event-free survival in prespecified subgroup.

	Total	CDKN2A/B deletion	CDKN2A/B wild type	HR (95% CI)	*p*-value	*p*-value for interaction
Number	81	37 (30.6)	44 (9.2)	4.148 (2.628–6.547)	<0.0001	
Gender						0.9523
Male	52	22 (34.9)	30 (10.5)	3.729 (2.149–6.472)	<0.0001	
Female	29	15 (25.8)	14 (7.3)	3.817 (1.842–7.910)	0.0003	
Age (years)						0.8944
<10	58	23 (27.4)	35 (7.9)	3.480 (2.056–5.891)	<0.0001	
≥10	23	14 (37.8)	9 (12.5)	3.260 (1.409–7.541)	0.0057	
Leukocyte counts						0.7870
<50×10^9^/L	47	16 (20.5)	31 (7.6)	3.003 (1.641–5.495)	0.0004	
≥50×10^9^/L	34	21 (48.8)	13 (26.4)	2.599 (1.301–5.192)	0.0068	
CNS status at diagnosis						0.2641
CNS1	66	27 (26.2)	39 (9.1)	3.121 (1.910–5.101)	<0.0001	
CNS2/traumatic lumbar puncture	14	9 (52.9)	5 (10.2)	6.312 (2.111–18.874)	0.0010	
Risk stratification						0.7156
LR	34	12 (19.6)	22 (7.0)	3.265 (1.615–6.601)	0.0010	
IR	47	25 (41.7)	22 (13.6)	2.639 (1.487–4.683)	0.0009	
ETV6-RUNX1						0.9772
Negative	75	36 (37.1)	39 (11.1)	3.822 (2.427–6.019)	<0.0001	
Positive	6	1 (4.2)	5 (4.0)	0.903 (0.105–7.732)	0.9257	
BCR-ABL						
Negative	73	31 (29.2)	42 (9.2)	3.467 (2.179–5.515)	<0.0001	0.7067
Positive	8	6 (40.0)	2 (9.5)	4.555 (0.913–22.728)	0.0645	
TP53 deletion						0.0445
Negative	72	29 (26.1)	43 (9.4)	2.993 (1.868–4.796)	<0.0001	
Positive	9	8 (80.0)	1 (5.3)	24.080 (2.978–194.738)	0.0029	
Response to prednisone						0.9476
PGR	45	19 (21.8)	26 (6.9)	3.413 (1.888–6.171)	<0.0001	
PPR	36	18 (52.9)	18 (18.0)	3.317 (1.725–6.379)	0.0003	

## Discussion

In this study, we present a large retrospective study of pediatric ALL patients with *CDKN2A/B* deletions treated in a single center and demonstrated the adverse effect of *CDKN2A/B* deletions on clinical outcomes.

The prevalence of *CDKN2A/B* deletions in our study was 20.2%, which was higher than Agarwal’s research (19.8%) but lower than Sulong’s research (22.0%) (2, 5). Previous investigators have reported the characteristics and clinical impact of *CDKN2A/B* deletions in pediatric ALL, and the prognostic importance of *CDKN2A/B* deletions in pediatric ALL is still controversial (2, 4, 5, 10, 19). We conducted a comprehensive analysis of *CDKN2A/B* deletions in 599 pediatric B-ALL patients. Data from our cohort showed that *CDKN2A/B* deletions were associated with older age at diagnosis, higher white blood cell counts, and prominent hepatosplenomegaly, which were consistent with the previous studies (5).

Many researchers concluded that *CDKN2A/B* deletions in childhood ALL were associated with an increased probability of relapse and death (2, 9, 10, 19–21), whereas Kima et al. and Mirebeau et al. concluded that homozygous *CDKN2A/B* deletion was not a poor prognostic factor in childhood B-ALL (4, 11). In our study, *CDKN2A/B* deletion carriers had decreased endpoints for 3-year EFS and OS compared to *CDKN2A/B* wild-type patients. Furthermore, our data showed that patients with the biallelic deletions had a worse survival rate (3-year EFS: 71.3 ± 6.0% vs. 67.7 ± 7.0% vs. 89.2 ± 1.6%, *p* = 0.000; 3-year OS: 922.2 ± 3.4% vs. 85.2 ± 5.2% vs. 94.7 ± 1.1%, *p* = 0.025) than patients without biallelic deletions (**Figure S1**).

The long-term outcome of pediatric ALL in China is optimal, especially for low-risk patients, and the OS rate remains 97.8% (12). Notably, in our cohorts, low-risk patients with *CDKN2A/B* deletions had inferior outcomes (3-year OS: 93.2 ± 3.3% vs. 97.9 ± 0.9%) compared to low-risk patients without *CDKN2A/B* deletions (**Figures 2C, D**). The result indicated that *CDKN2A/B* deletion patients even stratified in the low-risk group urgently needed intensive chemotherapy. Furthermore, allo-HSCT (*n* = 14) has improved overall survival than chemotherapy alone did (*n* = 20) in relapse patients with *CDKN2A/B* deletions, though no significance was observed between the two groups (3-year OS: 85.7 ± 9.4% vs. 60.5 ± 11.9%, *p* = 0.192) (**Figure S2**).

Sulong et al. reported that pediatric ALL with *CDKN2A/B* deletions had recurrent cytogenetic abnormalities including high frequencies of *TCF/PBX1*, *BCR/ABL*, hyperdiploidy, and *KMT2A*, and low frequencies of *ETV6/RUNX1* compared to patients without *CDKN2A/B* deletions (5, 11). We conclude a similar result in which *CDKN2A/B* deletion carriers had a higher prevalence of *BCR/ABL* and a low prevalence of *ETV6/RUNX1*. However, we did not find ph+ patients had inferior outcomes than ph- patients in *CDKN2A/B* deletion groups; this is most likely due to the utilization of tyrosine kinase inhibitors (TKIs) in ph+ patients for the entire duration of ALL therapy (22, 23). Pfeifer et al. demonstrated that *CDKN2A/B* deletions were adverse despite allogeneic stem cell transplantation in adult Philadelphia chromosome-positive (Ph+) ALL (24, 25); further studies in a larger cohort of pediatric Ph+ ALL with *CDKN2A/B* deletions are needed.

In our cohort, patients with *CDKN2A/B* deletions were more frequently steroid-resistant, whereas they had a better MRD clearance on day 19 of induction therapy compared with patients without *CDKN2A/B* deletions. These results differed from a study by Braun who found higher MRD levels on day 15 of induction therapy in patients with *CDKN2A/B* deletions (26). The differences in MRD clearance may be related to the patients’ race, chemotherapy protocols, and examination methods. An earlier study demonstrated that *CDKN2A/B* deletions were associated with unfavorable outcomes independent of MRD level in adult patients (27). However, both MRD positive on day 46 of induction therapy and *CDKN2A/B* deletion were still independent poor indicators for childhood ALL patients in our study.

Our study also indicated that patients with *CDKN2A/B* deletion had the worst prognosis in the *TP53* deletion subgroup. A similar outcome was found by Delfau-Larue for mantle cell lymphoma; i.e., patients with both *CDKN2A/B* and *TP53* deletions had the worst prognosis (28). The *CDKN2A/B* gene controls the cell cycle through the P53-MDM pathway; thus, co-occurrence of *CDKN2A/B* deletions and *TP53* deletions might enhance the aggressiveness of disease by strongly increasing the self-renewal capacity of leukemia cells (29–31). The hypothesis needs to be proved in future research.

Several limitations exist in this study. First, because of the limited sample size and short follow-up duration in relapse patients with *CDKN2A/B* deletions, the role of allogeneic transplant in the treatment needs to be interpreted carefully. Second, for the same reason, we did not conclude whether there had been significant interactions between two *TP53* deletions and *CDKN2A/B* deletions in pediatric ALL. CDKN2A gene can control cell cycle through the P53-MDM pathway; it might be attributed to the fact that CDKN2A deletion cooperates with the *TP53* deletion to enhance the aggressiveness of the disease by strongly increasing the self-renewal capacity of leukemia cells, but there is no theory to support this hypothesis.

In conclusion, a significant proportion of pediatric ALL patients still experience relapse, particularly patients with both *CDKN2A/B* and *TP53* deletions despite the high survival rate of childhood ALL. MDM2-P53 targeted agents are still in experimental research. In addition, CDK4/6 inhibitors (e.g., palbociclib) combined with chemotherapy are in clinical trials for the management of pediatric patients with relapsed/refractory ALL (32, 33). In the future, a new therapeutic strategy (e.g., target drug) based on genetic events might be applied in some subtypes of pediatric ALL.

## Data Availability Statement

The original contributions presented in the study are included in the article/**Supplementary Material**. Further inquiries can be directed to the corresponding author.

## Ethics Statement

The protocols described in this study were approved by the Ethics Committee, Institute of Hematology and Blood Disease Hospital, CAMS & PUMC. Written informed consent to participate in this study was provided by the participants’ legal guardian/next of kin.

## Author Contributions

All authors listed have made a substantial, direct, and intellectual contribution to the work and approved it for publication.

## Funding

The work is supported by the National Nature Science Foundation of China grants 81770175 (YCZ), 81870131 (XZ), and 81670112 (XZ).

## Conflict of Interest

The authors declare that the research was conducted in the absence of any commercial or financial relationships that could be construed as a potential conflict of interest.

## Publisher’s Note

All claims expressed in this article are solely those of the authors and do not necessarily represent those of their affiliated organizations, or those of the publisher, the editors and the reviewers. Any product that may be evaluated in this article, or claim that may be made by its manufacturer, is not guaranteed or endorsed by the publisher.
