# Supplementary figures and images for: Childhood Acute B-Lineage Lymphoblastic Leukemia With CDKN2A/B Deletion Is a Distinct Entity With Adverse Genetic Features and Poor Clinical Outcomes
Source: Front Oncol. 2022 May 24;12:878098. doi: 10.3389/fonc.2022.878098 (PMC9195293; doi:10.3389/fonc.2022.878098)

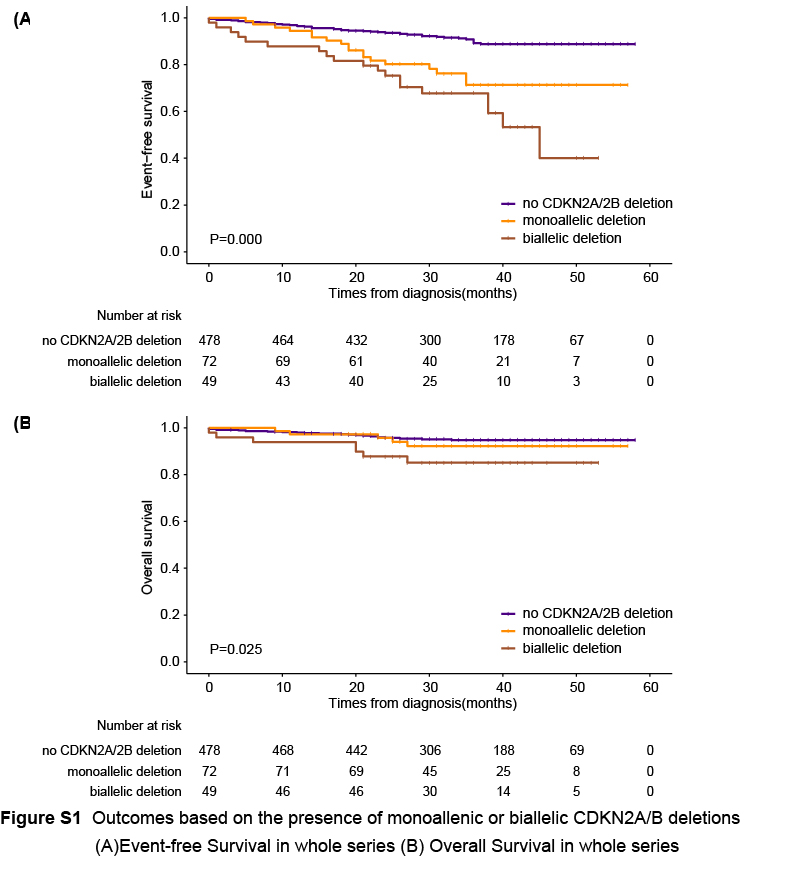

Supplement: Supplementary file 1 [file Image_1.jpeg]

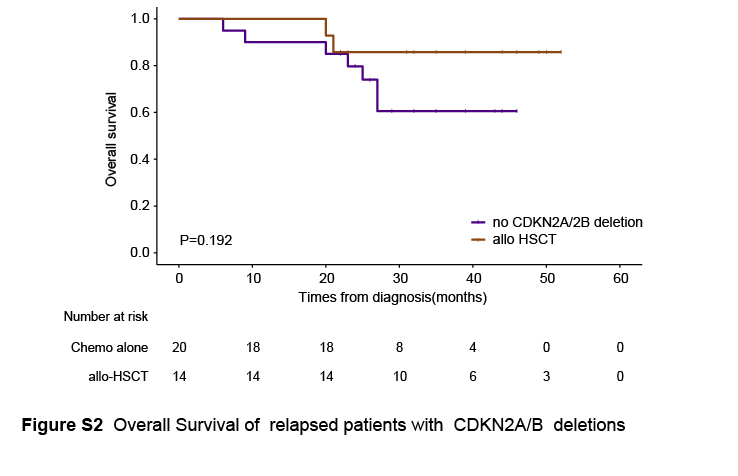

Supplement: Supplementary file 2 [file Image_2.jpg]
