# Supplementary material for: Childhood Acute B-Lineage Lymphoblastic Leukemia With CDKN2A/B Deletion Is a Distinct Entity With Adverse Genetic Features and Poor Clinical Outcomes
Source: Front Oncol. 2022 May 24;12:878098. doi: 10.3389/fonc.2022.878098 (PMC9195293; doi:10.3389/fonc.2022.878098)
Supplement: Supplementary file 3 [file Table_1.docx]

| **Table S1. Characteristics of patients with CDKN2A/B** **monoallelic or biallelic deletions** | | | |
| --- | --- | --- | --- |
| **Parameters** | **monoallelic deletions** | **biallelic deletions** | ***P*-value** |
| Number | 72 | 49 |  |
| Gender, male/female | 38/34 | 25/24 | 0.849 |
| Age, ≥10 years | 24(33.3) | 13(26.5) | 0.425 |
| Leukocyte counts, 10^9^/L(range) | 25.1(0.7-846.5) | 20.9(1.4-464.6) | 0.866 |
| Hepatosplenomegaly | 49(68.1) | 29(59.2) | 0.317 |
| CNS2/traumatic lumbar puncture | 8(11.1) | 9(18.4) | 0.260 |
| Risk stratification, IR | 36(50.0) | 24(51.0) | 0.912 |
| PPR | 19(26.4) | 15(30.6) | 0.612 |
| MRD positive at day 19 | 43(59.7) | 33(67.4) | 0.604 |
| MRD positive at day 46 | 13(18.1) | 9(19.2) | 0.881 |
| CR | 72(100.0) | 47(95.9) | 0.084 |
